# Co-feeding Transmission and Its Contribution to the Perpetuation of the Lyme Disease Spirochete *Borrelia afzelii* (In Reply to Randolph and Gern)

**DOI:** 10.3201/eid0907.030342

**Published:** 2003-07

**Authors:** Dania Richter, Rainer Allgöwer, Franz-Rainer Matuschka

**Affiliations:** * Humboldt-Universität zu Berlin, Berlin, Germany

**To the Editor:** Although transmission between co-feeding vector ticks may perpetuate particular tick-borne viruses, this mode of transmission plays no role in the epizootiology of Lyme disease spirochetes (*1*,*2*). In their letter, Randolph and Gern defend their suggestion that tick-borne pathogens perpetuate effectively by direct passage from one feeding tick to another by criticizing our analysis (*3*). These researchers mainly address our comparison of the transmission efficiency between simultaneously feeding ticks with that between ticks feeding sequentially on a persistently infected rodent. Our experiments demonstrate that approximately six times as many larvae (85.4%) acquire *Borrelia afzelii* spirochetes from a systemically infected mouse than from a mouse on which an infected nymph is feeding simultaneously (13.6%) (*1*). In nature, however, larval ticks rarely co-feed with nymphs on mice or voles; only approximately one fifth (18.8%) of these hosts harbor both subadult stages simultaneously. And of the nymphs, only approximately one quarter (26.4%) are infected by Lyme disease spirochetes. As a result, the natural transmission efficiency between simultaneously feeding ticks would be only one twentieth (5%) of that observed in the laboratory. Multiplying the experimentally observed efficiency of co-feeding transmission (13.6%) by the likelihood of larval and nymphal ticks co-infesting small rodents, as well as by the prevalence of infected nymphal ticks, reduces the efficiency of co-feeding transmission in nature to <1%. Although Randolph and Gern commit several minor mathematical errors, their calculations support our argument that few larval vector ticks would acquire spirochetal infection directly from an infected nymph (*3*).

Randolph and Gern err, however, by applying the same mathematical modifications to the transmission efficiency by which larvae acquire spirochetes from a persistently infected host (*3*). Whereas the efficiency of co-feeding transmission observed in the laboratory must be modified to pay tribute to the rare event when larvae co-feed with an infected nymph in nature, the efficiency by which larvae acquire infection from a persistently infected host is independent of such limiting parameters. Because a competent rodent host remains infectious to larval ticks throughout its life, the proportion of hosts infested by particular subadult stages of the vector is irrelevant. Thus, the transmission efficiency on a persistently infected host is unchanged in the laboratory and the field. Almost 85.4% of larvae feeding on mice or voles in nature would, therefore, acquire spirochetal infection—far more than by co-feeding. We are correct in stating that natural transmission by sequentially feeding ticks is more efficient than transmission between co-feeding ticks.

Randolph and Gern suggest that we could have recorded the distance between the feeding ticks to clarify whether the increase from a 13.6% transmission efficiency between co-feeding ticks to a transmission efficiency of 85.4% from a persistently infected host is associated with the development of a systemic infection. Our experimental observation (Table 1 in our article [*1*]), as well as a study on the movement of spirochetes through their host’s skin (*4*), conclusively demonstrates that the increase in transmission efficiency is due to the progressive dissemination of spirochetes from the site of inoculation. The likelihood of a larva’s acquiring spirochetes from any site of its host’s skin increases with the passage of time since the infected nymph attached. To compare the two modes of transmission in terms of efficiency (Table 2 in our article [*1*]), we permitted the larvae to attach randomly to their rodent hosts, just as they would do in nature.

**Table 1:** Spirochetal infection in larval *Ixodes ricinus* ticks that fed on mice during the period of attachment of  a single *Borrelia afzelii*–infected nymph and that fed at specified distances from the infecting nymph^a^

Duration of nymphal attachment before larvae attached (days)	Distance between nymph and larvae (cm)	Infection in co-feeding larvae


No. examined	% infected

0	Nil	68	0
	1	83	0
	2	51	0
1	Nil	125	1.6
	1	74	0
	2	124	0
2	Nil	67	29.9
	1	87	5.7
	2	54	1.9
3	Nil	94	55.3
	1	82	25.6
	2	160	6.3


^a^Each feeding sequence was replicated three times.

In the epizootiology of Lyme disease spirochetes, “simultaneous” transmission between co-feeding ticks (<1%) is some two orders of magnitude less efficient than sequential transmission between ticks feeding on persistently infected reservoir rodents (85.4%). The two studies that relied on natural infestation densities and refrained from using artificial feeding capsules conclusively demonstrated that transmission of Lyme disease spirochetes between ticks feeding simultaneously and in close proximity contributes little to the perpetuation of this pathogen, either in North America or in Europe (*1*,*2*). We are correct in concluding that Lyme disease spirochetes are maintained in nature mainly by sequential attachment of ticks to persistently infected reservoir hosts.

**Table:** **Table 2.** Spirochetal infection in larval *Ixodes ricinus* ticks that fed randomly on bodies of mice beginning at 3 days and 14 days after a single *Borrelia afzelii*–infected nymph had begun to feed




Duration of nymphal attachment before larvae attached (days)	Infection in larvae


No. examined	% infected

3	88	13.6
14	82	85.4
